# Muscle-Liver Substrate Fluxes in Exercising Humans and Potential Effects on Hepatic Metabolism

**DOI:** 10.1210/clinem/dgz266

**Published:** 2019-12-11

**Authors:** Chunxiu Hu, Miriam Hoene, Peter Plomgaard, Jakob S Hansen, Xinjie Zhao, Jia Li, Xiaolin Wang, Jens O Clemmesen, Niels H Secher, Hans U Häring, Rainer Lehmann, Guowang Xu, Cora Weigert

**Affiliations:** 1 CAS Key Laboratory of Separation Science for Analytical Chemistry, Dalian Institute of Chemical Physics, Dalian, China; 2 Institute for Clinical Chemistry and Pathobiochemistry, University Tuebingen, Tuebingen, Germany; 3 Department of Clinical Biochemistry, Rigshospitalet, Blegdamsvej, Copenhagen, Denmark; 4 The Centre of Inflammation and Metabolism and the Centre for Physical Activity Research, Department of Infectious Diseases and CMRC, Rigshospitalet, Blegdamsvej, Copenhagen, Denmark; 5 Department of Clinical Medicine, Faculty of Health and Medical Sciences, University of Copenhagen, Blegdamsvej, Copenhagen, Denmark; 6 Department of Hepatology, Rigshospitalet, Blegdamsvej, Copenhagen, Denmark; 7 Department of Anaesthesiology, The Copenhagen Muscle Research Centre, Rigshospitalet, Blegdamsvej, Copenhagen, Denmark; 8 Institute for Diabetes Research and Metabolic Diseases of the Helmholtz Zentrum Muenchen at the University of Tuebingen, Otfried-Mueller-Strasse, Tuebingen, Germany; 9 German Center for Diabetes Research (DZD), Ingolstädter Landstrasse, Oberschleissheim, Germany

**Keywords:** Capillary electrophoresis, metabolomics, succinate, exercise, liver, cAMP

## Abstract

**Context:**

The liver is crucial to maintain energy homeostasis during exercise. Skeletal muscle-derived metabolites can contribute to the regulation of hepatic metabolism.

**Objective:**

We aim to elucidate which metabolites are released from the working muscles and taken up by the liver in exercising humans and their potential influence on hepatic function.

**Methods:**

In two separate studies, young healthy men fasted overnight and then performed an acute bout of exercise. Arterial-to-venous differences of metabolites over the hepato-splanchnic bed and over the exercising and resting leg were investigated by capillary electrophoresis- and liquid chromatography-mass spectrometry metabolomics platforms. Liver transcriptome data of exercising mice were analyzed by pathway analysis to find a potential overlap between exercise-regulated metabolites and activators of hepatic transcription.

**Results:**

During exercise, hepatic O_2_ uptake and CO_2_ delivery were increased two-fold. In contrast to all other free fatty acids (FFA), those FFA with 18 or more carbon atoms and a high degree of saturation showed a constant release in the liver vein and only minor changes by exercise. FFA 6:0 and 8:0 were released from the working leg and taken up by the hepato-splanchnic bed. Succinate and malate showed a pronounced hepatic uptake during exercise and were also released from the exercising leg. The transcriptional response in the liver of exercising mice indicates the activation of HIF-, NRF2-, and cAMP-dependent gene transcription. These pathways can also be activated by succinate.

**Conclusion:**

Metabolites circulate between working muscles and the liver and may support the metabolic adaption to exercise by acting both as substrates and as signaling molecules.

For several decades, research has focused on the working skeletal muscle to understand how the muscle can achieve the pronounced increase in substrate oxidation that is necessary to provide adenosine triphosphate (ATP) for muscle contraction and movement. However, physical exercise is not possible without orchestrated cooperation of several tissues to support muscular work and to maintain metabolic homoeostasis. The central organ in this energy-demanding condition is the liver. Hepatic metabolism is crucial for glucose and lipid homoeostasis, for the recycling of metabolites and for the detoxifying of metabolic waste (1). Knowledge of the hepatic contribution to metabolism during acute exercise is mainly based on mouse studies and only a few human exercise studies (2). In humans, collection of blood samples from the hepatic vein and peripheral arteries has been employed to analyze arterial-to-hepato-splanchnic differences of selected metabolites and thus assess their hepato-splanchnic fluxes during exercise. These studies verified the exercise-induced increase in hepatic glucose output, the increased uptake of the gluconeogenic precursors lactate, pyruvate, and glycerol, of some amino acids, and of palmitate and oleate (3–5). The change in the hepato-splanchnic substrate fluxes is dependent on the duration and intensity of the exercise bout (6). In a recent study, we expanded the knowledge base on exercise-regulated metabolite fluxes over the hepato-splanchnic bed by performing targeted liquid chromatography-mass spectrometry (LC-MS) analysis of acylcarnitines in the hepatic vein, the femoral vein of the exercising and the resting leg, and peripheral arterial samples (7). Exercise amplifies the release of medium-chain acylcarnitines from the exercising leg and the uptake in the hepato-splanchnic bed, reflecting the huge increase in fatty acid oxidation in the working muscle and the function of the liver to buffer excess circulating metabolites by their uptake and conversion. In contrast, the liver constantly releases C2- and C3-carnitine during exercise, indicating an excess production of acetyl-CoA and propionyl-CoA (7).

All these data suggest that exercise is an energy-demanding metabolic challenge for the liver that goes along with the need for increased ATP production to maintain biosynthesis of substrates and recycling of metabolites. Consistent with this, analyses of hepatic tissue of mice after one acute bout of exercise revealed a pronounced change in the hepatic energy state with a drop in ATP, a huge increase in adenosine monophosphate (AMP), and activation of the AMP-activated kinase (8, 9). Transcriptome analyses in mice revealed that this is accompanied by profound alterations in hepatic transcript levels related to adaptive responses in glucose and fatty acid metabolism (10). Notably, studies on the release of hepatokines from the liver during or immediately after exercise in humans mirror the exercise-induced increase of the respective transcripts in exercising mice (11).

In humans, collection of hepatic tissue biopsies for molecular investigations before and immediately after exercise is not feasible. But by applying 2 complementary metabolomics analyses, capillary electrophoresis time-of-flight mass spectrometry (CE-TOF/MS) and ultra-high-performance liquid chromatography–quadruple-time-of-flight mass spectrometry (UHPLC-Q-TOF/MS), we can obtain a global picture of exercise-induced changes in the hepatic release or uptake of metabolites and thereby deepen our understanding of the hepatic contribution to exercise metabolism. First, we analyzed the metabolome in arterial and hepatic vein samples obtained at 8 time points during and after a 120-minute aerobic exercise trial at 60% VO_2_max to identify metabolites exhibiting exercise-induced changes. Next, we investigated which of these metabolites are released or taken up by the exercising skeletal muscle in order to obtain information on substrate fluxes between liver and muscle. When compared with the transcriptional response observed in livers of exercising mice, our data indicate that substrates delivered from the working muscle may not only support hepatic metabolism, but also contribute to the regulation of signaling pathways and gene transcription in the liver in response to exercise.

## Methods and Materials

### Experimental design

This work consists of 2 separate exercise studies to assess metabolite fluxes over the hepato-splanchnic bed and over the resting and exercising leg. The studies have been described in detail previously (12, 13), were executed in accordance with the Helsinki Declaration and were approved by the Scientific Ethics Committee of the capital region of Denmark. Subjects participating in the 2 studies were not identical. All subjects provided written informed consent to participate. In both studies, the participants were recreationally active and asked to refrain from strenuous exercise 24 hours before the experimental day. Blood was collected in EDTA-containing tubes, placed on ice, and immediately spun. Plasma aliquots were stored at −80^o^ C until analysis.

In the hepato-splanchnic exercise, 10 healthy men (age, 22.9 ± 0.8 years and body mass index [BMI], 22.6 ± 0.5 kg/m^2^) reported to the laboratory after an overnight fast (12). Catheters were inserted into the brachial artery of the nondominant arm and into a liver vein via the right femoral vein. The subjects performed a 2-hour cycling exercise at 60% VO_2_max in semi-supine position and remained fasting throughout the whole experimental period. Blood samples were obtained in pairs at each time point. Hepatic blood flow was measured by indocyanine green clearance (12). Samples obtained at baseline (0), 60, 120, 150, 180, 240, 300, and 360 minutes were used for metabolomics analysis. Samples for blood gas analysis were taken every 15 minutes throughout exercise and every 15 or 30 minutes during the recovery period. The content of oxygen (O_2_) and carbon dioxide (CO_2_) were measured using an ABL (Radiometer, Copenhagen, Denmark). Whole blood content of CO_2_ was calculated as described in (14) and whole blood O_2_ content was calculated by addition of O_2_ bound to hemoglobin with O_2_ dissolved in plasma. Finally, rates of O_2_ uptake and CO_2_ release were calculated by multiplying the arterial-to-venous difference with the hepatic blood flow.

The effect of exercise on metabolite fluxes over the leg was studied by analyzing plasma samples from a one-legged knee extensor study where the exercising leg was compared to the other (resting) leg (13). Briefly, after an overnight fast, 9 healthy young men (age, 20.9 ± 0.5 years and BMI, 22.6 ± 0.8 kg/m^2^) performed continuous one-legged knee-extensor exercise for 2 hours at 50% of maximum workload on a modified Krogh ergometer while the contralateral leg was resting. Catheters were inserted retrogradely into both femoral veins as well as into one femoral artery. The femoral retrograde placement avoids a contribution from the veins draining the lower abdominal adipose tissue (vena epigastrica superficialis) and leg cutaneous and subcutaneous tissues (vena saphena magna), which is important for the study of lipid metabolism (15). The participants were fasted until 3 hours after the exercise bout. Blood flow was determined for both the resting and the exercising leg using ultrasound Doppler in 3 subjects and the mean value was used to calculate net skeletal muscle release/uptake for all subjects. Three blood samples were drawn simultaneously at each time point.

### Chemicals and reagents

HPLC- or MS-grade solvents acetonitrile (ACN), methanol (MeOH), and chloroform (CHCl_3_) were purchased from Merck (Darmstadt, Germany) or Sigma-Aldrich (Steinheim, Germany or St. Louis, MO). MS grade water or ultrapure water was purchased from Merck (Darmstadt, Germany) or using a Milli-Q purity system (Millipore, Billerica, MA).

MeOH-dissolved internal standard solution (ISS-1) containing L-methionine sulfone, D-camphor-10-sulfonic acid sodium salt, leucine-d3, phenylalanine-d5, tryptophane-d5, succinic acid-13C4, and cholic acid-d4 (all from Sigma-Aldrich, Steinheim, Germany) was used to normalize signal intensity of the CE-TOF/MS data. ACN-dissolved ISS-2 consisting of N,N-diethyl-2-phenylacetamide, trimesic acid, disodium 3-hydroxynaphthalene-2,7-disulfonate (all from Wako, Japan), and 3-aminopyrrolidine dihydrochloride (Aldrich, St. Louis, MO) was used to adjust the migration time of the CE-TOF/MS analysis. Internal standards of free fatty acid (FFA) 16:0-d4 (Cambridge Isotope Laboratories, Tewksbury, MA, USA), and FFA 22:0-d4 (ten Brink laboratories, Amsterdam, Netherlands) dissolved in CHCl_3_/MeOH (2:1, v/v) were used to normalize signal intensity of the UHPLC-Q-TOF/MS-based FFA analysis.

### Sample preparation

Metabolite extraction for CE-TOF/MS-based metabolomics analysis was performed as previously described (16). Briefly, 50 µL of plasma was mixed with 450 μL MeOH solution containing ISS-1, followed by the addition of 500 μL of CHCl_3_. Subsequently, 200 μL of water was added, and samples were vortexed and centrifuged to form a 2-phase system. 420 μL of the upper layer was further centrifugally-filtered through a 5-kDa cutoff filter (HMT, Japan) for 2.5 to 3 hours at 4°C to remove residual proteins. The filtrate was lyophilized and stored at −80°C for later analysis. Prior to CE-TOF/MS metabolomics analysis, dry samples were preconditioned in Milli-Q water containing 50 μM ISS-2. Extraction and deproteination for UHPLC-Q-TOF/MS analysis of FFA not covered by CE-TOF/MS was performed according to a previously published protocol (17), using 100 μL of plasma and 300 μL of ACN.

### Metabolomics analysis

#### Capillary electrophoresis time-of-flight mass spectrometry.

CE-TOF/MS analysis was performed on a CE system (G7100A, Agilent, Santa Clara, CA)-TOF/MS (G6224A, Agilent) equipped with an ESI-MS sprayer kit (G1607A, Agilent), a 1260 ISO pump (G1310B, Agilent) and a minichiller (Huber, Offenburg, Germany). A fused-silica capillary with 80 cm × 50 μm internal diameter was used for sample analysis. The capillary temperature was maintained at 20°C. The sample tray temperature was set at 5°C controlled by the minichiller. The CE-TOF/MS coupling was realized by a coaxial sheath liquid interface. The sheath liquid containing MeOH/water (1:1, v/v) and 0.1 μM hexakis (2, 2-difluoroethoxy) phosphazene was delivered at 10 μL/min. CE-TOF/MS data acquisition of plasma samples was carried out in both cation and anion mode. The detailed parameters of these two scan modes were previously described (16). FFA containing fewer than 12 carbon atoms were analyzed by CE-TOF/MS.

#### Ultra-high-performance liquid chromatography–quadruple-time-of-flight mass spectrometry.

FFA containing 12 or more carbon atoms were analyzed using a Waters ACQUITY-UHPLC system (Waters Corp, Milford, USA) coupled to AB SCIEX Triple Q TOF 5600 plus System (AB SCIEX, Framingham, USA) operated in negative ion mode as previously described (18) with slight modifications. The separation was performed on a 2.1 × 100 mm ACQUITY 1.8 µm T3 column (Waters, Milford, MA, USA) and the mobile phase consisted of 6.5 mM ammonium bicarbonate in water (A) and 6.5 mM ammonium bicarbonate in 95% MeOH and water (B). The gradient elution started at 98% eluent A and was maintained for 1 minute, then linearly changed to 100% eluent B within 18 minutes and maintained for 4 minutes, and finally reverted back to 98% B and equilibrated for 2 minutes. Flow rate was 0.35 mL/min, and the column temperature was kept at 55°C. The ion spray voltage was set to 4500 V. Interface heater temperature was 500°C. Curtain gas, ion source gas 1 and ion source gas 2 were set to 35 PSI, 50 PSI and 50 PSI, respectively. FFA concentrations are given in µmol/L, concentrations of metabolites analyzed by CE-TOF/MS are given in arbitrary units (AU)/L.

#### Calculation of metabolite fluxes.

Hepato-splanchnic and leg fluxes (negative = release or positive = uptake) of a metabolite (a) were calculated by multiplying the arterial-to-venous difference by the plasma flow:

$$\begin{array}{ll} Flux_{a}\left(\frac{nmol}{min}\right)= & \left\{{\left[a\right]}_{artery}\left(\frac{nmol}{L}\right)-\;{\left[a\right]}_{vein}\left(\frac{nmol}{L}\right)\right\}\\ x\;Plasma\;flow\left(\frac{L}{min}\right) \end{array}$$

### Pathway analysis of liver transcriptome data of mice

Upstream regulator analysis using Ingenuity Pathway Analysis (Qiagen, Redwood City, CA) was performed with recently published whole genome array data of liver tissue of exercising male C57BL/6N mice (GEO database at NCBI; GSE110747) (19). Data of the control group of chow-fed mice were used for analysis. Livers were obtained immediately after 1 hour of treadmill running (13 m/min and 14 uphill slope) (19). Transcripts with a limma t-test *P* value < 0.05 and median fold change > |1.5| between exercised mice and sedentary mice were included in the analysis.

### Statistics

To identify metabolites with plasma concentrations affected by exercise on a systemic level, a one-way analysis of variance (ANOVA) was performed on arterial samples from the liver study (with time as fixed and subject as random effect). A Benjamini-Hochberg correction was performed with a false discovery rate (FDR) of 1%. A heatmap from the resulting subset of data was generated by unsupervised hierarchical clustering, using the open-source MultiExperiment Viewer software (20) and employing unit variance (UV)-scaled, mean-centered data.

Differences in metabolite flux over the hepato-splanchnic bed were detected using one-way ANOVA (time as fixed and subject as random effect) followed by Bonferroni post hoc test. Flux data were UV-scaled for the heatmap since mean-centering could lead to loss of directional information. The subset of metabolites exhibiting a significantly different hepato-splanchnic flux over time according to one-way ANOVA was further assessed in the one-leg exercise study, employing two-way ANOVA (time and resting/exercising leg flux as fixed, subject as random effect) followed by Bonferroni post hoc test.

Statistical analyses were performed using JMP 13.0 (SAS Institute Inc, Cary, NC). A *P* value < 0.05 was considered statistically significant. Data are presented as mean ± standard error (SEM).

## Results

### Increase in hepato-splanchnic oxygen consumption during exercise

**Figure 1.**
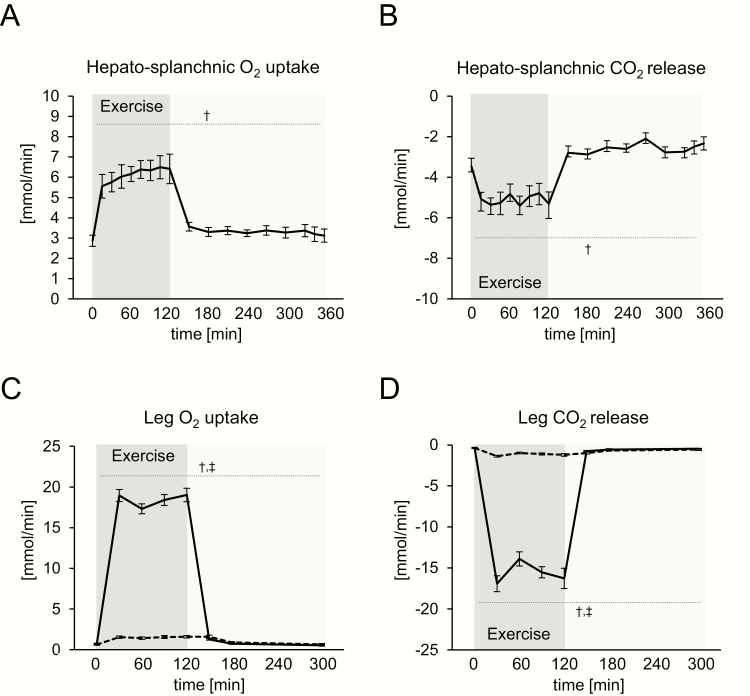
Blood gas fluxes in the hepato-splanchnic exercise study (1A, 1B) and one-legged knee extensor study (1C, 1D). 1A: Oxygen (O_2_) uptake and 1B: Carbon dioxide (CO_2_) release over the hepato-splanchnic bed. 1C: O_2_ uptake and 1D: CO_2_ release over the exercising (solid line) and resting leg (dashed line). Data are presented as mean ± standard error (SEM). † significant effect of time, ‡ significant effect of leg*time.

O_2_ uptake and CO_2_ release over the hepato-splanchnic bed were significantly increased immediately after the commencement of exercise and remained at this elevated level until the end of the exercise bout (Fig. 1A and 1B) with a similar hepato-splanchnic blood flow before (1.81 L/min) and during exercise (1.68 L/min). Before exercise, O_2_ uptake and CO_2_ release from the hepato-splanchnic bed were 3 mmol/min. The mean O_2_ uptake over the hepato-splanchnic bed during exercise was 6 mmol/min, paralleled by a CO_2_ release of 5 mmol/min. In comparison, O_2_ uptake of the exercising leg in the one-legged exercise study increased about 30-fold and reached a mean of 18 mmol/min during exercise, paralleled by a huge increase in CO2 release (Fig. 1C and 1D). Only marginal changes in O_2_ uptake and CO_2_ release of the resting leg were observed during the study. Before exercise at rest, the leg had a 3-fold lower O_2_ uptake than the hepato-splanchnic bed (1 vs 3 mmol/min).

### Exercise induces pronounced changes in the arterial concentration of metabolites

**Figure 2.**
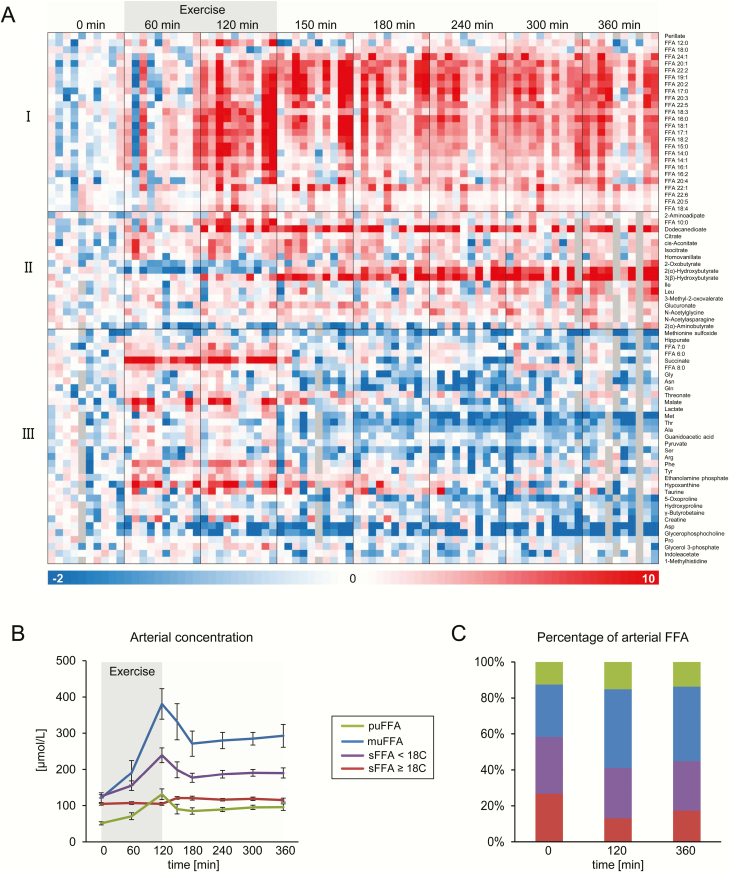
Effects of exercise on systemic concentrations of metabolites detected in plasma using CE-TOF/MS and UHPLC-Q-TOF/MS in the hepato-splanchnic exercise study. 2A: Metabolites significantly affected by exercise on the arterial (systemic) level, clustered by Spearman rank-order correlation. Each column represents unit variance (UV)-scaled, mean-centered values from 1 individual. 2B: concentration (mean ± standard error [SEM]) and 2C: percentages of free fatty acids (FFA), summed up according to chain length and degree of saturation (Abbreviations: mu, mono-unsaturated; pu, poly-unsaturated; s, saturated FFA). Only FFA with a chain length of more than 10 carbon atoms, from the UHPLC-Q-TOF/MS analysis, were included.

CE-TOF/MS metabolomics analyses were performed in order to analyze a broad range of metabolites in the blood samples. Fatty acids with a chain length of more than 10 carbon atoms were quantified by UHPLC-Q-TOF/MS. The arterial plasma concentration of 77 of the over 200 detected metabolites was significantly changed (one-way ANOVA with FDR < 1%) during the exercise trial (Fig. 2A). Grouping of metabolites by Spearman rank-order correlation resulted in 3 main clusters (Fig. 2A). Cluster I contains metabolites that were increased at the end of the exercise bout and remained elevated in the recovery phase, in particular FFA. The metabolites in cluster II are characterized by an increase in the recovery phase, while their concentration was unchanged or even dropped during exercise. In this cluster, 2-(α)-hydroxybutyrate and 3-(β)-hydroxybutyrate continuously increased until the end of the recovery phase. The participants stayed fasted after exercise, which could explain the still-elevated concentration of many metabolites in the recovery phase. The metabolites in cluster III showed an early and transient increase during exercise, and/or a decline in the recovery phase compared to pre-exercise values. Succinate, malate, lactate, hypoxanthine, FFA 6:0, FFA 7:0, and FFA 8:0 were grouped into this cluster since they were increased during the exercise bout.

### Exercise increases the proportion of monounsaturated FFA in plasma

Exercise did not cause an equal increase in the arterial plasma concentration of all FFA species. Immediately at the end of the exercise bout, there was no observed increase in saturated FFA with a chain length of 18 and more carbon atoms (Fig. 2B). This resulted in an increase in the proportion of several mono- and polyunsaturated FFA, which were elevated after exercise. This effect was particularly pronounced for the abundant monounsaturated FFA species (29% ± 6% before vs 44% ± 4% after exercise; Fig. 2C), while the proportion of the saturated FFA with 18 and more carbon atoms was reduced (27% ± 5% before vs 13% ± 3% after exercise).

### Exercise regulates the hepato-splanchnic flux of metabolites

**Figure 3.**
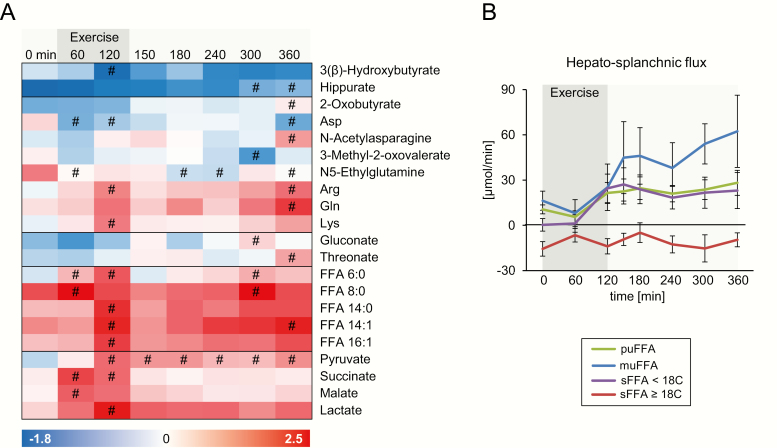
Effects of exercise on the hepato-splanchnic flux of metabolites. 3A: Metabolites exhibiting significant changes in uptake (positive values, red) or release (negative values, blue) from the hepato-splanchnic bed. Each column represents unit variance (UV)-scaled values of the mean of all participants. Metabolites were ordered according to pathway and kinetics. 3B: Hepato-splanchnic flux of free fatty acids (FFA) quantified by UHPLC-Q-TOF/MS, summed up according to chain length and saturation (Abbreviations: mu, mono-unsaturated; pu, poly-unsaturated; s, saturated FFA) presented as mean ± standard error (SEM). #: significantly different from 0 minutes.

The hepato-splanchnic flux of all detected metabolites was calculated as the difference between the concentrations of arterial and hepatic vein sample multiplied by hepatic blood flow. During the exercise trial, the hepato-splanchnic flux of 21 metabolites was significantly altered at one or more timepoints (one-way ANOVA with Bonferroni post hoc test). The flux of these metabolites is illustrated as a heatmap (Fig. 3A) with the red color indicating an uptake and the blue color indicating a release from the hepato-splanchnic bed. Exercise increased the uptake of the medium-chain FFA 6:0 and 8:0 and of the long-chain FFA 14:0, 14:1, and 16:1 at 60 and/or 120 minutes. The alterations in the uptake followed the changes in the arterial concentrations of individual FFA. Again, saturated FFA with 18 carbon atoms and more differed from the other FFA species because they showed a constantly negative hepato-splanchnic flux, indicating a release from the hepato-splanchnic bed (Fig. 3B). This opposite regulation of long-chain and very-long-chain saturated FFA was also observed before exercise in the fasting state (Table 1).

**Table 1.** FFA > 10 carbon atoms exhibiting hepato-splanchnic uptake or release in the basal, fasting state

	Hepato-splanchnic flux [µmol/min]		t-test against 0
	Mean	SEM	*P*-value
FFA 17:0	-0.19	0.08	0.0499
FFA 18:0	-15.15	4.71	0.0106
FFA 20:0	-0.23	0.09	0.0263
FFA 24:0	-0.06	0.02	0.0454
FFA 24:1	-0.03	0.01	0.0157
FFA 12:0	0.73	0.17	0.0018
FFA 14:0	2.53	0.52	0.0009
FFA 14:1	0.51	0.09	0.0003
FFA 16:1	4.41	0.99	0.0016
FFA 16:2	0.06	0.01	0.0005
FFA 17:1	0.15	0.04	0.0046
FFA 18:2	7.24	1.99	0.0055
FFA 18:3	1.26	0.22	0.0003
FFA 18:4	0.05	0.01	0.0049
FFA 22:5	0.35	0.06	0.0004
FFA 22:6	1.24	0.49	0.0315

Negative values indicate a hepato-splanchnic release, positive values an uptake. The flux was assessed by a t-test against 0 (no uptake/release), a *P* < 0.05 was considered significant

An increased uptake during or after exercise was also observable for succinate, malate, lactate, and pyruvate, and for the amino acids arginine, glutamine, and lysine (Fig. 3A). Threonate and N-acetylasparagine only showed an uptake after 360 minutes. Of all these metabolites, only succinate and FFA 8:0 were significantly taken up in the fasting state (Table 2). A release from the hepato-splanchnic bed during and/or after exercise was detected for 3-(β)-hydroxybutyrate, hippurate, aspartate, 3-methyl-2-oxovalerate, and N5-ethylglutamine.

**Table 2.** Metabolites analyzed by CE-TOF/MS exhibiting hepato-splanchnic uptake or release in the basal, fasting state

	Hepato-splanchnic flux [AU/min]		t-test against 0
	Mean	SEM	*P*-value
2-(α)-Hydroxybutyrate	-4.06	1.64	0.0430
2-Oxobutyrate	-1.56	0.35	0.0029
3-(β)-Hydroxybutyrate	-20.40	7.17	0.0249
3-Phenylpropionic acid	-0.33	0.11	0.0174
Citrulline	-16.41	5.08	0.0120
Glutamate	-19.17	4.92	0.0045
Hippurate	-2.23	0.55	0.0050
Isobutyrate	-2.79	1.09	0.0373
N2-Phenylacetylglutamine	-0.23	0.06	0.0082
N-Acetylalanine	-0.31	0.12	0.0444
Propionate	-1.34	0.41	0.0133
trans-Cinnamate	-0.77	0.12	0.0004
1-Methyladenosine	0.04	0.01	0.0273
2-Hydroxy-4-methylvaleric acid	0.10	0.03	0.0116
5-Oxoproline	1.83	0.40	0.0026
Alanine	22.65	6.70	0.0096
Asparagine	3.42	1.37	0.0367
Benzoate	0.48	0.15	0.0169
Citrate	9.91	2.68	0.0077
FFA 8:0	2.10	0.43	0.0017
FFA 10:0	1.24	0.32	0.0064
Guanidoacetic acid	1.35	0.35	0.0050
Methionine	2.62	1.11	0.0456
N5-Ethylglutamine	0.45	0.19	0.0459
Succinate	0.79	0.16	0.0017

Negative values indicate a hepato-splanchnic release, positive values an uptake. The flux was assessed by a t-test against 0 (no uptake/release), a *P* < 0.05 was considered significant.

### Increase in substrate fluxes between skeletal muscle and liver during exercise

**Figure 4.**
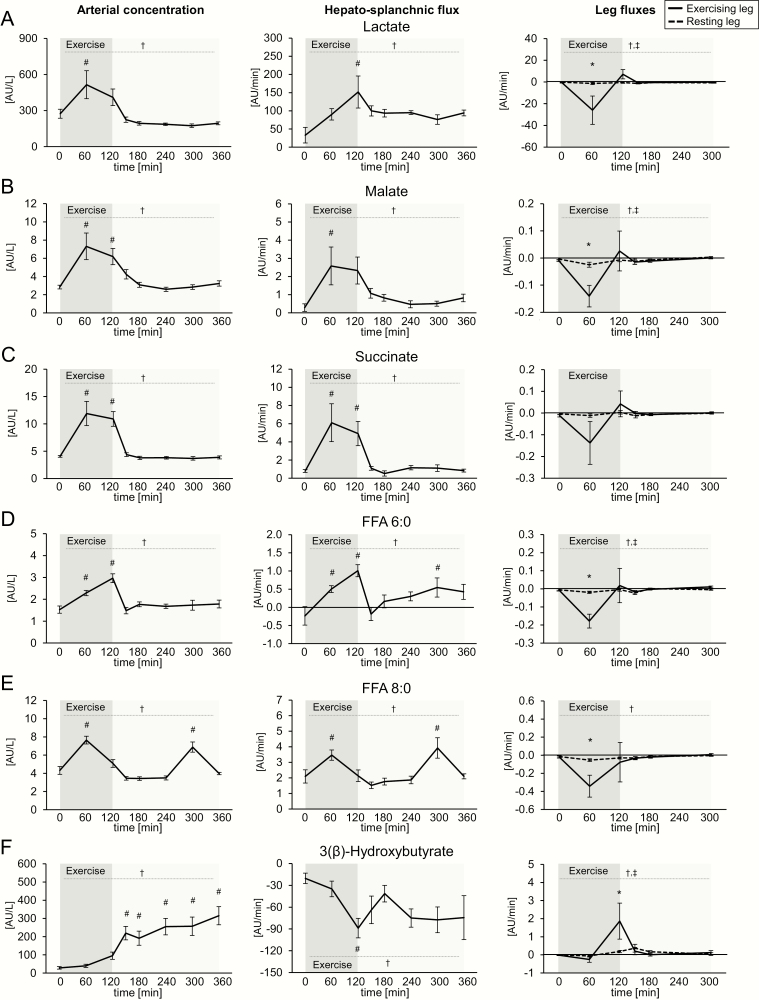
Metabolites exhibiting a significant increased uptake into or release from the hepato-splanchnic bed were further assessed for an opposing flux from the exercising leg. This was the case for A: lactate, B: malate, C: succinate, D: FFA 6:0, E: FFA 8:0 and F: 3-(β)-hydroxybutyrate. Shown are arterial (systemic) concentrations from the hepato-splanchnic exercise study (left panel), hepato-splanchnic fluxes (middle panel) and fluxes over the resting and exercising leg (right panel). Data are presented as mean ± standard error (SEM). †: significant effect of time; ‡: significant effect of leg*time; #: significantly different from 0 min; *: significantly different between exercising and resting leg according to Bonferroni post-hoc test. Succinate showed a significant difference between exercising and resting leg after 60 minutes of exercise, but only a trend towards a time effect in the two-way ANOVA (*P* = 0.0945).

Next, we studied which of the metabolites with exercise-induced changes in the hepato-splanchnic flux showed an oppositely changed flux over the exercising leg compared with the resting leg in the one-legged exercise study. This was, as expected, the case for lactate, which was released from the exercising leg after 60 minutes of exercise (Fig. 4A). The exercising leg also contributed to the systemic increase of malate, succinate, FFA 6:0 and 8:0, which may support the uptake into the hepato-splanchnic bed (Fig. 4B–E). Thus, these metabolites may circulate from the contracting muscle to the liver. In particular, citric acid cycle (TCA cycle) metabolites malate and succinate showed similar exercise-regulated kinetics of their arterial concentration and fluxes with a pronounced hepatic uptake after 60 and 120 minutes of exercise (Fig. 4B,C). In contrast, the liver released 3-(β)-hydroxybutyrate. An uptake of 3-(β)-hydroxybutyrate by the exercising leg was observed in the one-legged exercise study (Fig. 4F).

### Fatty acids, cyclic AMP, hypoxia-induced factor 1A, and nuclear factor erythroid-2-related factor 2 are identified as upstream regulators of the hepatic transcriptional response to exercise

Metabolites can be shuttled into metabolic pathways, but also act as signaling molecules: by binding to membrane or nuclear receptors and to intracellular proteins, or as substrate for protein modifications with impact on transcriptional activation and epigenetic regulation of gene expression (21–23). In order to investigate which of the metabolites with an increased hepato-splanchnic uptake during exercise are potentially implicated in the regulation of hepatic transcripts, we analyzed the acute transcriptional response of the liver to exercise. Since the acute regulation of hepatic transcripts is hardly accessible in humans, we made use of data from exercising mice recently published by our group (19). Pathway analysis of the exercise-regulated transcripts indicated activation of peroxisome proliferator-activated receptor (PPAR), glucocorticoid-activated receptor signaling, and of interleukin 6-, p53- and GADD45-dependent signaling (Tables 3, 4), which is in accordance with previous data (10). Transcription data also indicated pronounced cyclic AMP (cAMP)-dependent activation of transcription factors (CREB, CREM, and FOXO) and activation of the hypoxia-induced factor (HIF)1A, as well as activation of nuclear factor erythroid 2-related factor 2 (NRF2)-dependent transcription. Fatty acids and cAMP were among the identified endogenous substances implicated in the transcriptional activation of genes (Table 4).

**Table 3.** Canonical pathways with enriched exercise-regulated transcripts in the liver of mice after a 1h-treadmill run

Canonical pathway	*P*-value
PPAR Signaling	6.17E-04
IL-6 Signaling	1.55E-03
PI3K Signaling in B Lymphocytes	2.24E-03
p53 Signaling	3.89E-03
Glucocorticoid Receptor Signaling	5.25E-03
IGF-1 Signaling	5.37E-03
SPINK1 General Cancer Pathway	6.92E-03
PXR/RXR Activation	7.24E-03
GADD45 Signaling	7.24E-03
NRF2-mediated Oxidative Stress Response	7.59E-03
Thrombopoietin Signaling	9.55E-03

Ingenuity canonical pathway analysis of transcripts that were significantly different (limma t-test *P* < 0.05 and median fold change > |1.5|) between exercised and sedentary mice immediately after a 1h treadmill run (GSE110747 (19), only canonical pathways with a *P*-value < 0.01 are shown).

**Table 4.** Upstream regulators implicated in the activation of genes in the liver of mice after a 1h-treadmill run

Transcriptional regulators			Endogenous substances		
Name	Z-score	*P*-value	Name	Z-score	*P*-value
CREB1^*^	4.14	1.27E-15	hydrogen peroxide	3.438	1.74E-09
RELA	3.40	6.55E-09	Ca2+	3.098	6.10E-13
FOXO3^*^	3.21	5.01E-10	leukotriene D4	3.095	2.10E-12
HIF1A^*^	2.95	1.63E-03	cyclic AMP^*^	2.878	5.32E-08
FOXO1^*^	2.94	6.85E-10	prostaglandin E2	2.588	1.06E-12
STAT3	2.85	7.35E-11	norepinephrine	2.53	4.55E-11
TP63	2.75	1.79E-04	corticosterone	2.414	1.13E-05
NFKBIA	2.72	3.32E-08	fatty acids	2.387	5.18E-06
NFKB1	2.60	6.96E-06	histamine	2.189	1.26E-04
SMAD3	2.59	5.32E-08	nitric oxide	2.173	3.25E-10
CREM^*^	2.50	3.83E-10			
TP53	2.49	1.33E-06			
FOXL2	2.44	2.94E-06			
CTNNB1	2.43	2.62E-03			
MYOD1	2.37	2.88E-04			
MEF2C	2.37	2.87E-06			
NUPR1	2.33	6.17E-03			
CDKN2A	2.32	1.38E-08			
MEF2D	2.20	5.93E-05			
ELK1	2.19	1.70E-06			
EGR1	2.18	4.98E-06			
PDX1	2.17	3.94E-11			
NFYA	2.16	2.29E-05			
NRF2^*^	2.06	3.81E-06			
STAT6	2.00	4.19E-05			

Ingenuity upstream regulator analysis of transcripts that were significantly different (limma t-test *P* < 0.05 and median fold change > |1.5|) between exercised and sedentary mice immediately after a 1h treadmill run (GSE110747; (19)). Regulators with z-score > 2.0 (ie, predicted to be activated) and with a p-value of overlap <0.05 are shown (other regulator groups are not included). ^*^Transcription regulators discussed as succinate-regulated factors are formatted bold.

## Discussion

In this study we provide, for the first time to our knowledge, a comprehensive metabolomics analysis of human plasma samples collected at 8 time points from the artery and the hepatic vein during and after an acute bout of exercise. The participants performed aerobic endurance exercise at 60% VO_2_max for 120 minutes. At this intensity, hepatic blood flow was not reduced and an increase in hepatic oxygen uptake was observed. An approximately 2-fold increase of oxygen uptake has also been reported in other studies performed at a moderate exercise intensity, such as during exercise at 30% VO_2_max for 240 minutes (3), at 55% VO_2_max for 60 minutes (24), and at 60% VO_2_max for 120 minutes (25). Even with a 50% reduction in hepatic blood flow during intense exercise, a slightly increased oxygen uptake was reported (6), which underlines the capacity of the hepato-splanchnic region to increase oxygen extraction in order to maintain metabolic processes during states of blood flow redistribution towards the working muscle (26). Together with the increase in CO_2_ release, this is a clear indication of higher metabolic activity in the liver of exercising humans and underlines an increased demand for ATP as reported in the liver of exercising mice (8).

An important ATP-consuming metabolic process in the liver during exercise is the production of glucose from glucogenic precursors. In the overnight fasted participants of our study, net hepato-splanchnic glucose production increased from 1 mmol/min to approximately 3 mmol/min (12). Gluconeogenesis accounts for 20% to 25% of total glucose production when exercise is performed after an overnight fast at 45% or 65% of VO_2_peak, conditions similar to our study (27). Increased hepato-splanchnic uptake of the glucogenic substrates lactate, pyruvate, and lipolysis-derived glycerol during exercise has been reported previously (4, 28). By our CE-TOF/MS, which also covered polar metabolites, we confirmed the exercise-induced increase in the hepato-splanchnic uptake of lactate and pyruvate. The exercise-dependent regulation of glycerol was not detectable with our approach since the filters used for sample preparation contain glycerol, which cannot be completely washed out and cannot be distinguished from plasma glycerol. Two of the amino acids that exhibited an increased hepato-splanchnic uptake during the trial, arginine and glutamine, are glucogenic. Other glucogenic amino acids such as alanine showed already at baseline, in the fasting state, a positive value for the hepato-splanchnic flux and thus an uptake (Table 2). The findings demonstrate that our approach is suitable to analyze substrate fluxes over the hepato-splanchnic bed.

A constant finding of our study is that the chain length and degree of saturation has a strong influence on the hepato-splanchnic exchange of FFA during exercise, but also in the baseline condition after the overnight fast. Similar data have been reported earlier, with high hepatic uptake rates for oleate (FFA 18:1), linoleate (FFA 18:2), laureate (FFA 12:0) and myristate (FFA 14:0) during exercise (29), while stearate (FFA 18:0) has a negative arterial-hepatic vein difference during exercise (5). The broad coverage of FFA by UHPLC-Q-TOF/MS-based metabolomics in our study makes a more systematic interpretation possible. In general, FFA with at least 18 carbon atoms and a high degree of saturation showed a negative hepato-splanchnic flux at baseline, and only a marginal increase in the arterial concentration after exercise. The net release of FFA from the hepato-splanchnic bed is presumably due to fatty acid delivery from intraabdominal adipose tissue (4), and these adipose depots contain higher proportion of long-chain saturated fatty acids compared with subcutaneous depots (30). The increase in the systemic concentration is partly due to an overspill of fatty acids from exercise-induced lipolysis of subcutaneous adipose tissue, which contains higher proportions of oleate and linoleate compared with plasma concentrations but less stearate (31). Thus, the different increase in the arterial concentration of these fatty acids reflects the adipose depot-specific fatty acid composition of triglycerides. As a consequence, a higher proportion of unsaturated FFA was found in plasma after exercise compared with pre-exercise values, which is in accordance with previous reports (32). The data also indicate that the contribution of other nonhepatic intraabdominal tissues to the measured hepato-splanchnic fluxes must be considered.

A novel finding is the efflux of FFA 6:0 and FFA 8:0 from the exercising muscle and their increased uptake into the hepato-splanchnic bed. Since the respective C6:0 and C8:0 acylcarnitine esters are strongly increased in exercising muscle tissue (14- and 8-fold, respectively) (7), fatty acid chain shortening during β-oxidation followed by hydrolysis of acylcarnitine esters is a likely explanation for the increased release from the exercising leg. Medium-chain fatty acids are considered as easily accessible fuels, since they can be metabolized independent of proteins for binding and transport (33). Isolated liver perfusion studies or incubation of isolated hepatocytes show an enhanced mitochondrial respiration, a glucose-sparing effect towards fatty acid oxidation and a stimulation of gluconeogenesis, in particular by FFA 8:0 (34, 35). An intriguing idea worth studying further is the potential relevance of muscle-derived FFA 6:0 and 8:0 for the adaption of hepatic metabolism to exercise.

The main rationale for employing the CE-TOF/MS approach in our study was to track the flux of a wide range of polar metabolites, which are not covered by common LC-MS–based metabolomics methods. In addition to the known substrate flux of lactate from the exercising muscle to the liver and the flux of 3-(β)-hydroxybutyrate from the liver to the exercising muscle, our data revealed the uptake of malate and succinate into the hepato-splanchnic bed during exercise and their release from the exercising leg. There was no or only a small detectable uptake before exercise (Table 2) suggesting that the pronounced increase in arterial plasma is the driver of the increased uptake. Arterial concentrations and fluxes of malate and succinate showed similar kinetics during the study. Both are intermediates of the TCA cycle, and succinate is converted to malate via succinate dehydrogenase (SDH), which catalyzes the reduction to fumarate and fumarase, which catalyzes the hydration to malate. Since SDH couples the TCA cycle to the respiratory chain, succinate is an electron donor and driver of oxidative phosphorylation, but it is also substrate for protein modifications and has signaling and paracrine/endocrine effects (36). Succinate has structural similarity to 2-ketoglutarate and can compete for the binding to 2-ketoglutarate-dependent dioxygenases (2-KGDD), causing inhibition of these enzymes (37). One prominent target of 2-KGDD is the transcription factor HIF, which was activated in the livers of exercising mice according to transcriptome analysis (Table 4). The inhibition of these enzymes by succinate causes stabilization of HIF, increased activation of HIF-target genes (38), and links the accumulation of succinate to HIF-induced interleukin 1beta expression (39). High fumarate concentrations cause S-(2-succino)-cysteine modifications of proteins, a process called succination (40). The succination of Kelch-like ECH-associated protein-1 (KEAP)1 releases the transcription factor NRF2 from its complex with KEAP1 and enables its nuclear translocation and activation of target genes (41). The transcription factor NRF2 was also identified as activated in the livers of exercising mice (Tables 3, 4). Another mechanism for how elevated succinate concentrations could influence hepatic signaling pathways was described recently in mice, where the succinate-induced HIF activation leads to accumulation of cAMP by inhibition of its degradation (42). Notably, cAMP is a master regulator of the transcriptional response to exercise in the liver, which is responsible for the activation of the CREB1, CREM, and FOXO transcription factors (Table 4). Thus, accumulation of succinate has been linked to the activation of certain transcription factors and to cAMP-dependent transcriptional activation, and these succinate-dependent pathways share a great overlap with the pathways which are found to be acutely activated in the liver of mice after exercise (43). This overlap remains speculative without experimental validation. Moreover, we performed this comparison of metabolomics with transcriptomics data across different species, which can be seen as another limitation. But human transcriptome data obtained from liver tissue under exercise conditions are hardly accessible. In conclusion, we suggest that succinate can be a candidate for the list of metabolic upstream regulators of the hepatic exercise response, such as fatty acids as PPAR activators and the glucagon/insulin ratio as cAMP inducer. Our data are a first hint that succinate accumulation in hepatic tissue can support the acute transcriptional response to exercise. Notably, the more than 6-fold increase in the hepato-splanchnic uptake of succinate may cause tissue concentrations sufficient to inhibit 2-KGDD (44, 45).

It is intriguing to hypothesize that the working leg is a major source of the increased hepato-splanchnic uptake of the TCA metabolites, lactate, and FFA 6:0 and FFA 8:0. However, the metabolomics data were obtained from 2 different exercise studies, and a direct comparison is not possible since the exercise modalities are different. In particular, the ongoing hepato-splanchnic uptake in the second exercise phase after the leg release peaked at 60 minutes raises the question of other exercise-dependent sources of these metabolites. To the best of our knowledge, no human data are available to support the contribution of other tissues. It can be speculated that tissues with increased metabolic activity during exercise, such as the heart, can contribute with an increased net release of TCA metabolites. Up to now, the release of succinate from the heart was mainly reported during ischemic conditions (46). Another possible candidate is the adipose tissue, which is postulated to show increased net release of lactate during exercise (47).

## Conclusions

The application of a global metabolomics approach to investigate the effect of exercise on substrate fluxes offers the possibility to identify metabolites exchanged between tissues and to elucidate group-specific differences in regulation (eg, in the hepatic uptake or systemic increase of different FFA). Comparison of the known effects of the muscle-derived metabolites succinate and FFA 8:0 on hepatic metabolism and transcriptional regulation with the exercise-dependent regulation of hepatic metabolic pathways reveals a surprising overlap. The results underline the essential function of the hepatic metabolism during exercise and support the relevance of the crosstalk of working muscles and the liver to exchange metabolite substrates, but also signaling molecules, that support and mediate compensatory and adaptive processes during exercise.

## Abbreviations

2-KGDD: 2-ketoglutarate-dependent dioxygenase
ACN: acetonitrile
AMP: adenosine monophosphate
ANOVA: analysis of variance
ATP: adenosine triphosphate
BMI: body mass index
CE-TOF/MS: capillary electrophoresis-time of flight mass spectrometry
cAMP: cyclic AMP
CHCl_3_: chloroform
FDR: false discovery rate
FFA: free fatty acids
HIF: hypoxia-induced factor
HPLC: high-performance liquid chromatography
ISS: internal standard solution
KEAP1: kelch-like ECH-associated protein-1
LC-MS: liquid chromatography-mass spectrometry
MeOH: methanol
NRF2: nuclear factor erythroid-2-related factor 2
SDH: succinate dehydrogenase
TCA cycle: citric acid cycle (tricarboxylic acid cycle)
UHPLC-Q-TOF/MS: ultra-high-performance liquid chromatography–quadruple-time-of-flight mass spectrometry
UV: unit variance

## Additional Information

***Disclosure Summary:*** The authors have nothing to disclose.

***Data availability:*** All data generated or analyzed during this study are included in this published article or in the data repositories listed in References.
